# Development and feasibility of a personalized, interactive risk calculator for knee osteoarthritis

**DOI:** 10.1186/s12891-015-0771-3

**Published:** 2015-10-22

**Authors:** Elena Losina, Kristina Klara, Griffin L. Michl, Jamie E. Collins, Jeffrey N. Katz

**Affiliations:** Orthopaedic and Arthritis Center for Outcomes Research, Department of Orthopedic Surgery, Brigham and Women’s Hospital, 75 Francis St, BC 4-016, 02115 Boston, MA USA; Department of Biostatistics, Boston University School of Public Health, Boston, MA 02118 USA; Harvard Medical School, Boston, MA 02115 USA; Division of Rheumatology, Immunology and Allergy, Brigham and Women’s Hospital, Boston, MA 02115 USA

**Keywords:** Knee, Osteoarthritis, Knee OA, Risk calculator, OA risk

## Abstract

**Background:**

The incidence of knee osteoarthritis (OA) is rising. While several risk factors have been associated with the development of knee OA, this information is not readily accessible to those at risk for osteoarthritis. Risk calculators have been developed for several prevalent chronic conditions but not for OA. Using published evidence on established risk factors, we developed an interactive, personalized knee OA risk calculator (OA Risk C) and conducted a pilot study to evaluate its acceptability and feasibility.

**Methods:**

We used the Osteoarthritis Policy (OAPol) Model, a validated, state-transition simulation of the natural history and management of OA, to generate data for OA Risk C. Risk estimates for calculator users were based on a set of demographic and clinical factors (age, sex, race/ethnicity, obesity) and select risk factors (family history of knee OA, occupational exposure, and history of knee injury). OA Risk C presents personalized risk of knee OA in several ways to maximize understanding among a wide range of users. We conducted a study of 45 subjects in a primary care setting to establish the feasibility and acceptability of the OA risk calculator. Pilot study participants were asked several questions regarding ease of use, clarity of presentation, and clarity of the graphical representation of their risk. These questions used a five-level agreement scale ranging from strongly disagree to strongly agree.

**Results:**

OA Risk C depicts information about users’ risk of symptomatic knee OA in 5 year intervals. Study participants estimated their lifetime risk at 38 %, while their actual lifetime risk, as estimated by OA Risk C, was 25 %. Eighty-four percent of pilot study participants reported that OA Risk C was easy to understand, and 89 % agreed that the graphs depicting their risk were clear and comprehensible.

**Conclusions:**

We have developed a personalized, computer-based OA risk calculator that is easy to use. OA Risk C may be utilized to estimate individuals’ knee OA risk and to deliver educational and behavioral interventions focused on osteoarthritis risk reduction.

## Background

Knee osteoarthritis (OA) is a condition that affects the bones, cartilage, and other tissues in the knee and often leads to pain and physical limitation. While the severity of pain fluctuates during the course of the disease that lasts on average 26 years from the time of diagnosis, it is unlikely that symptoms disappear completely [1, 2]. The disease affects nearly 9.3 million US adults, accounting for $27 billion in annual health care expenses [3, 4]. While generally considered a disorder of aging, the incidence of knee OA in younger adults is rising, with about 50 % of diagnoses occurring in patients younger than 55 [5]. While not every radiographic change leads to the development of the symptomatic disease, those with symptoms often seek medical care and ultimately consider total knee replacement, due to limited non-surgical treatment options [6, 7].

A number of risk factors have been identified for knee OA. Older age, female sex, obesity, occupational exposure, and history of knee injury have been associated with the development of the disease [8–10]. With the greater obesity epidemic [11] and an increasing number of traumatic knee injuries [12], the clinical and economic burden of knee OA will likely continue to grow. In addition to accumulation of risk factors, aging of the population plays a key role in the increasing prevalence of knee OA. Despite the increasing incidence of OA and the substantial disability associated with the disease, to our knowledge, few studies have focused on primary prevention efforts [13–17]. While OA prevention across all ages is important, preventive interventions focused primarily on older populations may have a limited effect due to prolonged exposure to risk factors earlier in life. Primary prevention efforts that raise awareness of modifiable OA risk factors in younger populations may offer a greater impact.

Online risk calculators have been developed for several diseases, including heart disease, cancer, diabetes, stroke, emphysema, and osteoporosis [18–22]. These tools request that users enter demographic and risk factor information. The tools then provide an estimate of the user’s risk for developing a particular illness. The American Heart Association and American College of Cardiology have published online and mobile versions of their risk calculator for public use.

Websites hosting risk calculators are highly trafficked. In 2006, the *Your Disease Risk* website [23], which estimates visitors’ risk of developing seven different diseases, including cancer, diabetes, and heart disease, recorded 2000 visitors per day on average [24]. In a 2013 survey of over 3000 US adults, the Pew Research Internet Project found that 72 % of internet users had consulted online sources for health information in the past year [25]. To our knowledge, there is no online risk calculator for osteoarthritis. The availability of a risk assessment tool for OA would raise awareness of modifiable risk factors for the condition. We sought to develop an interactive, personalized, computer-based risk calculator for knee OA (OA Risk C). We tested the feasibility and acceptability of the calculator in a sample of OA-free patients in a primary care setting.

## Methods

### Risk calculator development and derivation of OA risk

We used data generated by the Osteoarthritis Policy (OAPol) Model to create a computer-based risk calculator for knee OA (OA Risk C). OA Risk C allows users to enter their demographic and risk factor information, including family history of osteoarthritis, exposure to occupational risk factors (such as squatting and kneeling), and history of knee injury. Users are matched to one of 2016 OAPol simulations, which provides an estimate of their 5, 10, 15, 20, 25, 30-year, and lifetime risk of knee OA and total knee replacement (TKR).

### Structure of the OAPol model

OAPol is a validated, published, state-transition, Monte Carlo model of the natural history and management of knee OA [5, 6, 26, 27]. “State transition” implies that the model follows each person’s history as a sequence of annual transitions from one health state to another. Health states are designed to be predictive of clinical prognosis related to knee OA, risk for developing comorbidities, and mortality. The OAPol Model considers five major comorbidities (cardiovascular disease, diabetes mellitus, cancer, chronic obstructive pulmonary disease, and other musculoskeletal diseases). The prevalence and incidence of each of these comorbidities are stratified by age, sex, race/ethnicity, and obesity. Prevalence of cardiovascular disease is further stratified by diabetes mellitus status. Persons who develop comorbidities have higher risk for death. The explicit accounting for competing risks from other comorbidities is a special feature of the OAPol model.

Each person is followed from the time of entry into the OAPol Model until death. Upon entry into the model, a person is randomly assigned age, sex, race/ethnicity, and body mass index (BMI) from a set of user-specified probability distributions. At the start of each annual cycle, the model records the patient’s age, obesity status (defined by BMI), symptomatic knee osteoarthritis status, and presence of specific comorbidities. The model then uses these characteristics, in addition to sex and race/ethnicity, to determine the probabilities that indicate development of symptomatic knee osteoarthritis, progression of symptomatic knee osteoarthritis, development of a new comorbidity, and death in the subsequent year. Transition probabilities are derived from literature or secondary analyses of data from national- or population-based cohorts and translated into risk functions for the model. Details of transitional probabilities derivation have been previously published [26]. Upon the person’s death, summary statistics are recorded and a new person enters the model. A large number of individual simulations are aggregated to obtain stable estimates of OA and TKR rates. The size of the hypothetical cohort, N, is selected to ensure stable population estimates. For the purposes of establishing stable estimates for risk of knee OA, we used *N* = 1,000,000 for each model run.

We conducted 2016 model simulations using each unique combination of seven input parameters. These included starting age (25–45), sex (male, female), race/ethnicity (White, Black, Hispanic), obesity status (obese, non-obese), family history of knee OA (present, absent), occupational exposure to OA risk (present, absent), and history of knee injury (present, absent) (Table 1).

**Table 1:** Model inputs

Input parameter	Possible values			
Starting Age	25 years through 45 years			
Sex	Female			
	Male			
Race/ethnicity	White			
	Black			
	Hispanic			
Obesity status	Obese			
	Non-obese			
Family history	Family history			
	No family history			
Occupational exposure	Present			
	Absent			
History of knee injury	Present			
	Absent			
*OA Incidence (annual probability, %)*				
Age Range	Non-obese male	Non-obese female	Obese Male	Obese Female
25–34	0.12	0.14	0.25	0.38
35–44	0.13	0.15	0.25	0.41
45–54	0.23	0.28	0.47	0.62
55–64	0.41	0.46	0.76	1.19
65–74	0.24	0.31	0.46	0.55
75–84	0.21	0.22	0.36	0.48
85+	0.21	0.22	0.36	0.48
*OA Progression Estimates (annual probability, %)*				
			Male	Female
Progression from K-L 2 to K-L 3 (non-obese/obese)			5.58/ 12.26	4.00/ 8.95
Progression from K-L 3 to K-L 4 (non-obese/obese)			1.29/ 2.94	1.95/ 4.27
*Risk Factors (multipliers for increased OA incidence, derived from Zhang* et al. [33]*)*				
Knee injury	2.39			
Family history of OA	1.72			
Occupational risks	1.28			

This table lists model input parameters and values used to create the 2016 simulations for OA Risk C

We defined incident knee osteoarthritis as Kellgren-Lawrence (K-L) grade 2 accompanied by knee pain. Base incidence rates were derived from the National Health Interview Survey and stratified by age, sex, and obesity. Details on incidence rates derivation have been previously published [28]. Upon development of symptomatic knee OA, subjects could progress through K-L grades 2 through 4 based on probabilities derived from the Johnston County Osteoarthritis Project, a cohort study of OA [29] and calibrated to published rates of annual knee OA progression [30]. Progression probabilities were stratified by sex, obesity, and current K-L grade (Table 1). Annual incidence of TKR was derived from two multi-centered longitudinal studies: MOST (Multicenter Osteoarthritis Study) and OAI (Osteoarthritis Initiative) [31, 32]. Further details of incidence rate derivation have been published elsewhere [7].

To account for increased risk of developing knee OA for those with a family history of knee OA, occupational exposure, obesity, or history of knee injury, we used the published data from Zhang et al. [33]. Based on the data from Zhang et al., knee injury increased OA incidence by a factor of 2.39. Family history of OA increased OA incidence by a factor of 1.72, and occupational risks by a factor of 1.28 (Table 1). Risk factors were assumed to be independent and multiplicative. As noted above, age, sex and obesity status were estimated directly based upon National Health Interview Survey data [28]. For example, to incorporate the impact of obesity, we first derived the incidence of knee OA among those who are not obese. This was done by dividing the age/sex stratified incidence of knee OA by the sum of the prevalence of obesity multiplied by the increased risk of OA due to obesity and prevalence of non-obesity. To ensure that we did not overinflate the risk of OA among obese persons, we applied increase in risk to the incidence of OA among those who are non-obese persons, derived as described above. We used the incidence rate among non-obese individuals as the basis for the risk adjustment for other risk factors.

### Risk calculator design and user interface

**Fig. 1:**
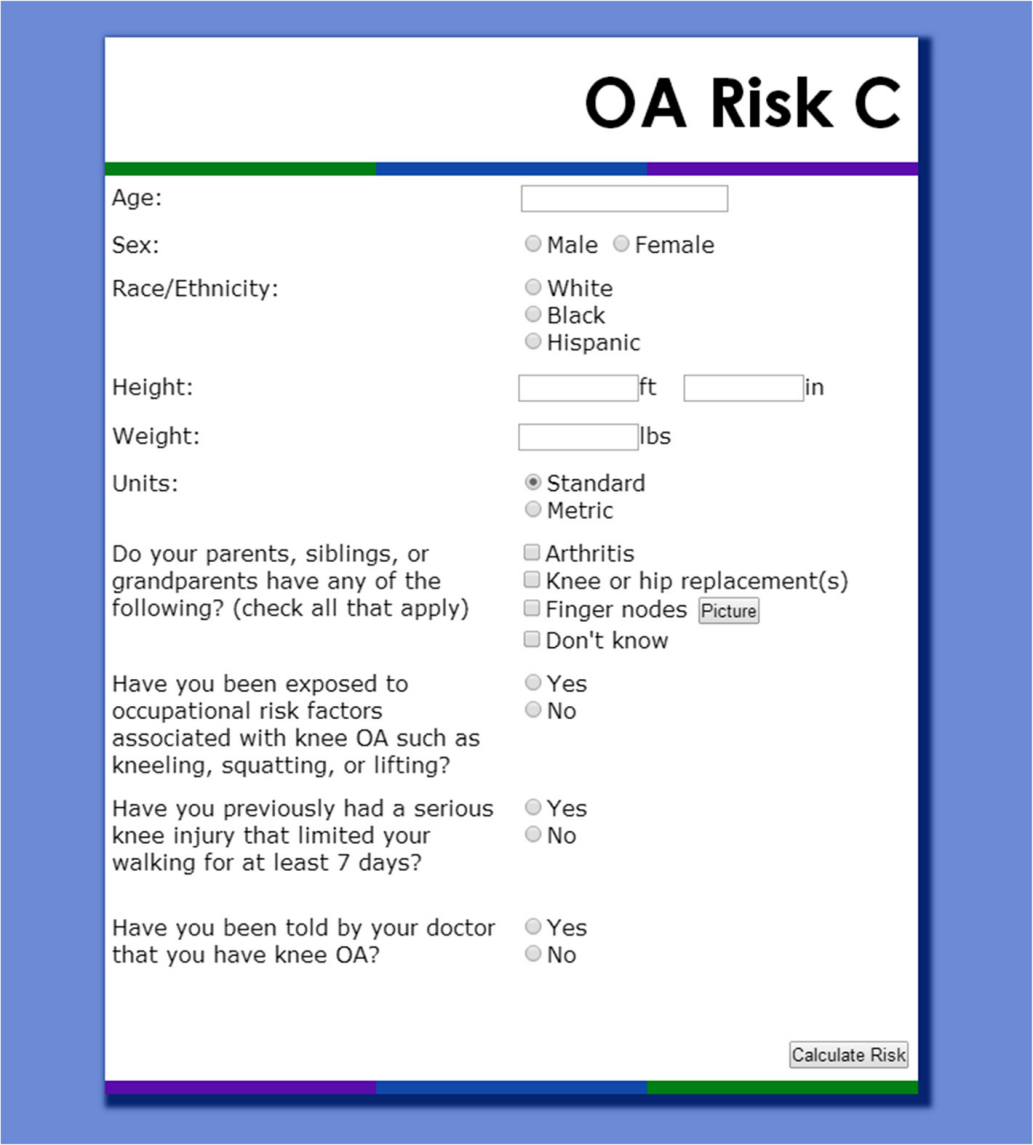
Risk Calculator User Inputs – This figure shows the first page of the Osteoarthritis Risk Calculator. Users enter their demographic and risk-related information, which the calculator cross-references against OAPol model outputs in order to determine each user’s individual risk of knee osteoarthritis and total knee replacement

OA Risk C users input demographic and risk factor information, including age, sex, race/ethnicity, height, and weight. Questions about risk factors are asked in such a way that someone unfamiliar with OA could answer them. Family history of osteoarthritis is determined by asking: “Do your parents, siblings, or grandparents have any of the following: Arthritis (osteoarthritis), Knee or hip replacement, Finger nodes?” Users are asked to check all that apply, including “Don’t Know” (Fig. 1). Users have the option of viewing a popup image of finger nodes if they are unfamiliar with the term. If the user selects any of the first three options (arthritis, knee or hip replacement, finger nodes), he or she is considered to have a family history of OA. Occupational risk is determined using the question: “Have you been exposed to occupational risk factors associated with knee OA such as kneeling, squatting, or lifting?” History of knee injury is determined by asking: “Have you previously had a serious knee injury that limited your ability to walk for at least 7 days?” This question conforms to the definition of knee injury used in the Osteoarthritis Initiative, a multi-center cohort study of knee OA [33].

**Fig. 2:**
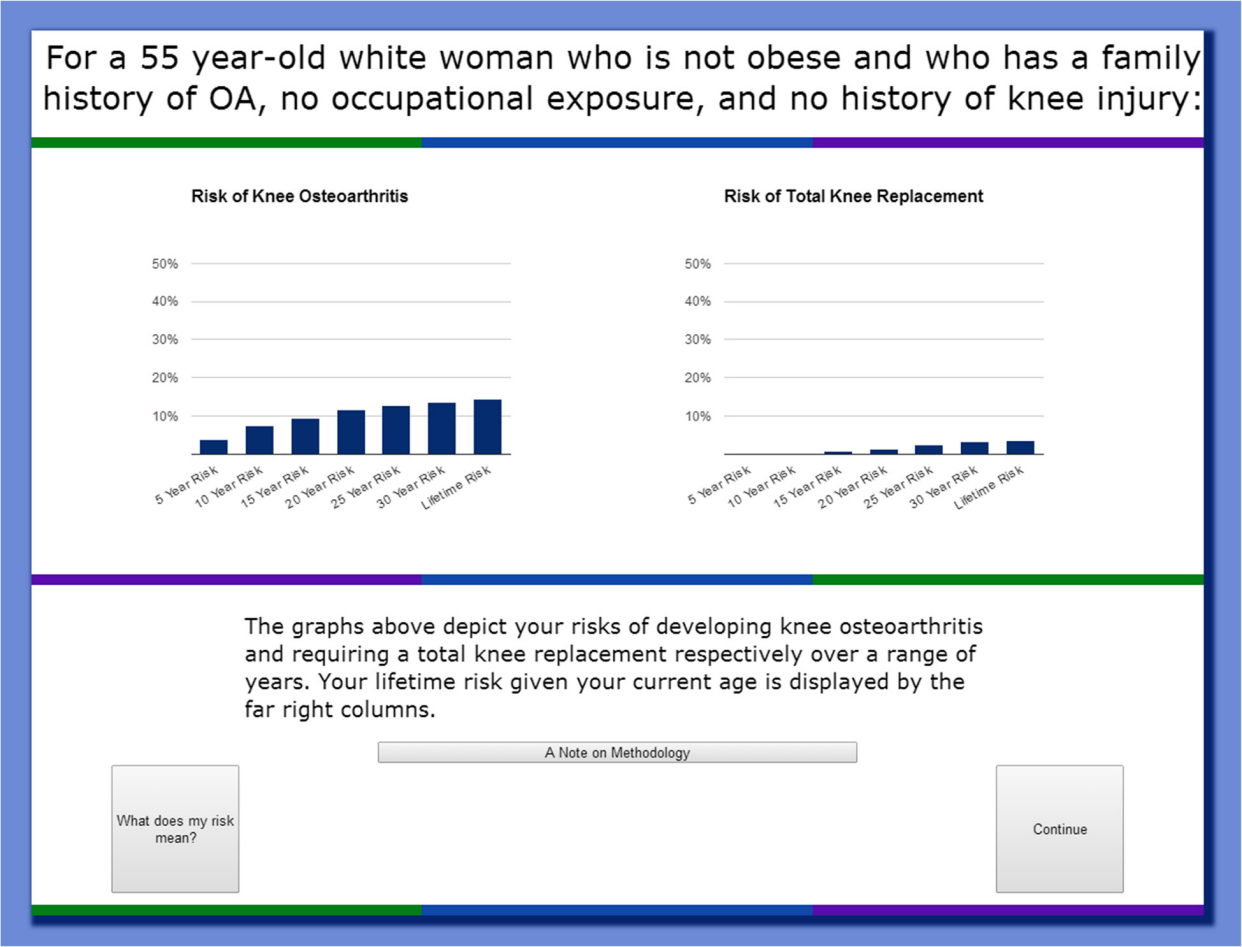
Graphical Risk Information – This figure shows an example of the first set of personal risk information provided to subjects in the form of bar graphs. In this example, we have included risk information for a 55 year-old white, non-obese woman with a family history of OA but with no occupational exposure or history of knee injury. OA Risk C users are presented with their 5-, 10-, 15-, 20-, 25-, 30-year and lifetime risk of knee OA and total knee replacement. By clicking “What does my risk mean?” users may view the same data in icon array form

**Fig. 3:**
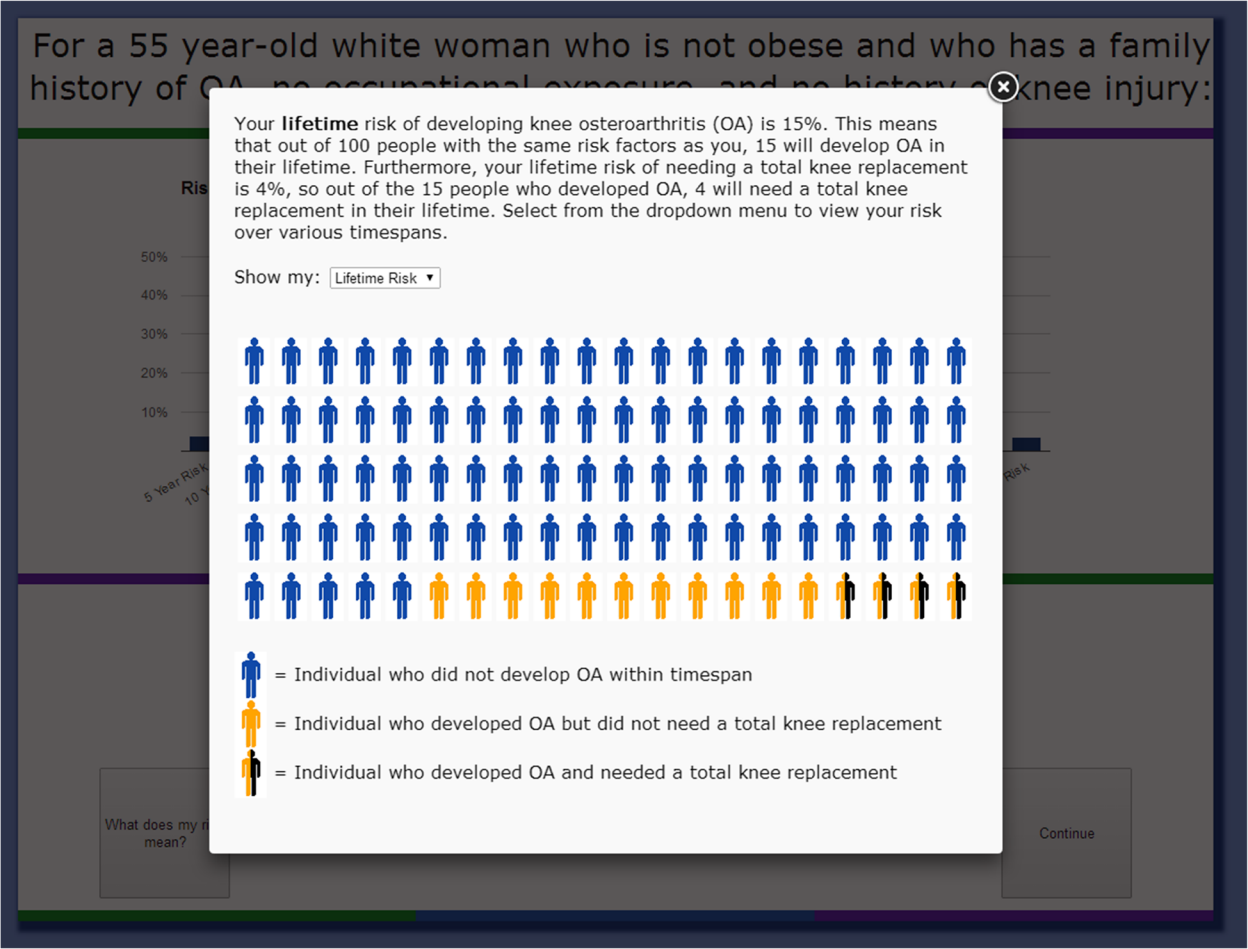
Icon Array Risk Information – This figure shows the pop up that appears when calculator users click “What does my risk mean?” Participants can click on the dropdown menu (“Show my:”) to select the timeframe (5-, 10-, 15, 20-, 25-, 30-year or lifetime) for which they want to view their risk in icon array form

**Fig. 4:**
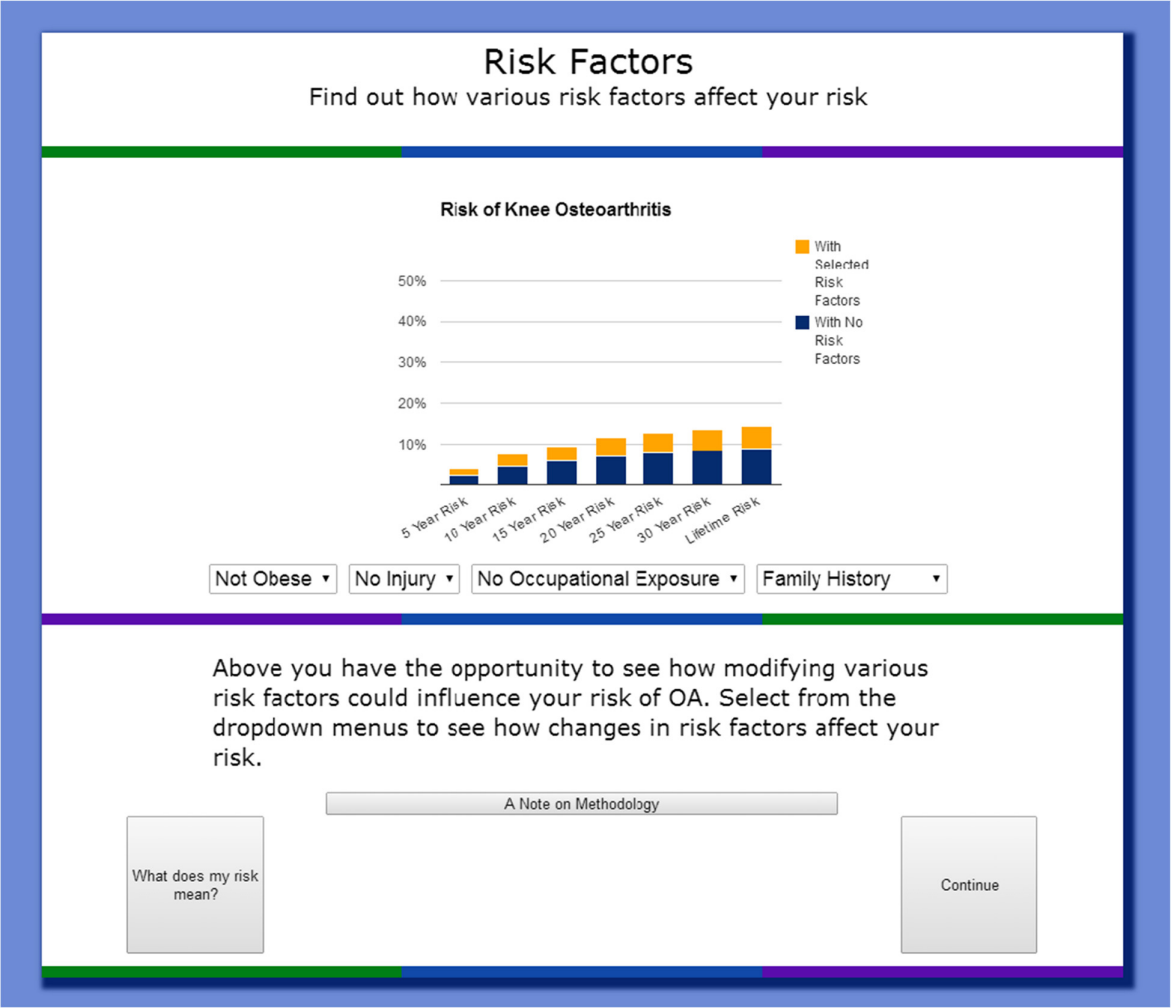
Interactive Graphical Risk Information – This figure shows the interactive page of the risk calculator. Users can select from dropdown menus to add or remove risk factors and observe how their risk changes. Users can also click “What does my risk mean?” in order to view the same information in icon array form

**Fig. 5:**
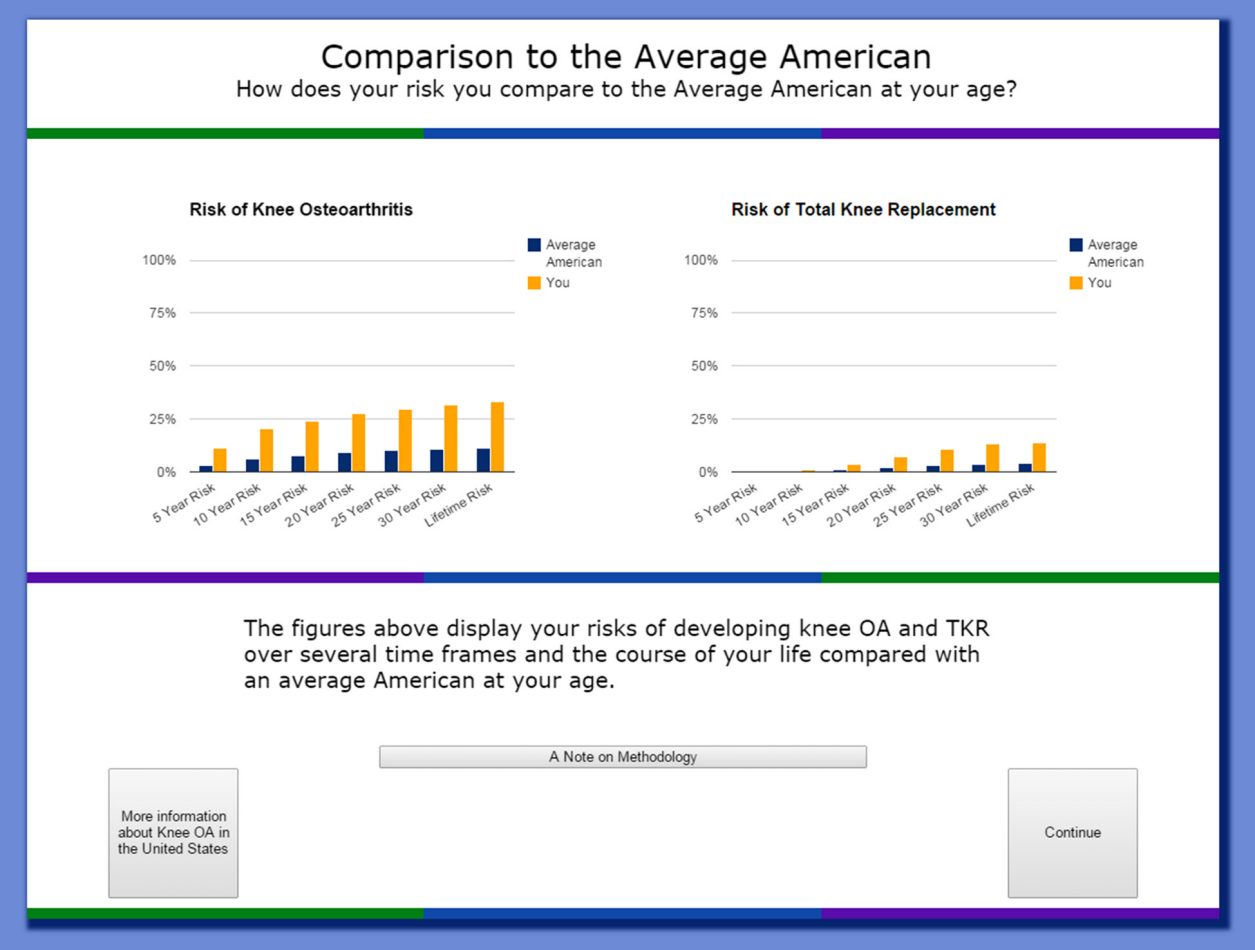
Comparison to the Average American – This figure shows the participant how their risk of developing knee OA or undergoing TKR compares to the Average American with no risk factors over several time frames (5-, 10-, 15-, 20-, 25-, 30-, and lifetime risk). The bar graphs show the user’s risk in yellow and the Average American’s risk in blue

Users are shown the results of their risk calculation in several different ways. The first page contains a simple graphical representation of their 5, 10, 15, 20, 25, 30-year, and lifetime risk of developing knee osteoarthritis and total knee replacement (Fig. 2). We also present risk information in icon array form, which users can access by clicking “What does my risk mean?” An example of this pictorial representation of the risk of developing knee OA is shown in Fig. 3. Then, users are taken to the interactive component of the risk calculator, where they can learn how different factors contribute to their risk of developing knee OA. Users are able to adjust their risk factors on an interactive graph. Four dropdown menus allow users to add or remove each risk factor and see how their risk assessment changes (Fig. 4). For example, a non-obese person can view their risk of developing osteoarthritis if they were obese. Users are also able to see which component of their risk is due to added risk factors, and which component simply reflects the underlying risk experienced by someone of their demographic group with no additional risk factors. As on the previous page, users are given the option to view this information in icon array form. Finally, users are shown a graphical comparison of how their personal risk of developing knee OA and undergoing TKR compares to that of the average American with no risk factors (Fig. 5).

### Pilot study of risk calculator clinical implementation

In order to determine the feasibility and acceptability of the OA Risk Calculator, we enrolled 45 subjects at a primary care clinic within a tertiary medical center. Both patients and non-patients in the waiting room of the clinic were invited to participate. Those who spoke English, were between the ages of 25 and 45 years, had not been diagnosed with arthritis by a physician, had access to a computer and the Internet, and were willing to provide their email address were eligible for the study. After agreeing to participate, eligible subjects read and signed a digital consent form. They were given a convertible laptop (which functions as both a laptop computer and tablet) and instructed to follow the directions on the computerized risk calculator until the completion page appeared. Because the aim of pilot testing was to determine the usability of the calculator, the research assistant administering the calculator asked subjects to refrain from commenting or asking questions until the end of the survey if possible. After using OA Risk C, we provided study participants with a brochure about OA that included suggestions for minimizing exposure to modifiable OA risk factors. Patients were given a 2-h parking voucher for their participation or an Amazon gift card of equivalent value ($9) if they did not park.

To measure feasibility and acceptability of the calculator, we asked subjects to rate its ease of use and comprehensibility on a 5-point agreement scale with the following options: Strongly Disagree, Disagree, Neutral, Agree, and Strongly Agree. Participants were also asked for suggestions for improvement and general feedback. This study was approved by the Partners Healthcare Institutional Review Board (protocol 2014P000844).

## Results

**Fig. 6:**
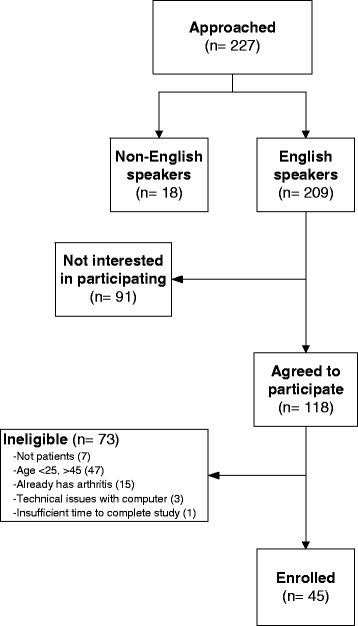
Pilot Study Recruitment – This figure shows the pilot study recruitment process. The number of subjects deemed eligible and ineligible to participate at each enrollment stage, along with reasons for ineligibility, are included

We asked 227 people in the clinic waiting room to take part in the study. Two-hundred and nine (92 %) were English-speakers, and of those, 118 (56 %) agreed to participate. Seventy-three (62 %) of those who agreed were ineligible: 7 were not patients at the clinic and were approached before we gained approval to recruit non-patients for the study; 47 were not between the ages of 25 and 45 years; 15 had been diagnosed with arthritis; 3 were excluded because of lapses in wireless internet access; and 1 was excluded because they had insufficient time to complete the study (Fig. 6).

Forty-five patients were enrolled and completed the OA Risk C assessment. Average age was 34 (standard deviation [SD] 6) years, and 87 % were females. Thirty-one percent identified themselves as White, 27 % as Black, and 36 % as Hispanic. Forty-six percent had a Bachelor’s degree or higher. Forty-four percent reported having a family member with arthritis; 36 % reported having an occupational exposure, shown to be associated with higher incidence of osteoarthritis; and 7 % reported history of knee injury (Table 2). More than one third of subjects (38 %) had at least two risk factors for knee OA, and about two thirds had at least one risk factor. On average, study participants overestimated their lifetime risk of developing knee OA. The average lifetime risk estimate was 38 %, while average lifetime risk as determined by the calculator was 25 %.

**Table 2:** Cohort characteristics

Parameter	Mean (SD) or N (%)
*Demographics*	
Age	34 (6)
BMI	29 (6)
Sex	
Male	6 (13 %)
Female	39 (87 %)
Race/ethnicity	
White	14 (31 %)
Black	12 (27 %)
Hispanic	16 (36 %)
Other	3 (7 %)
*Risk Factors*	
Family History	22 (49 %)
Injury	3 (7 %)
Occupation	16 (36 %)

This table shows the demographic and risk factor information for pilot study participants. Values for each parameter are presented either as number and percent of participants or mean and standard deviation

**Fig. 7:**
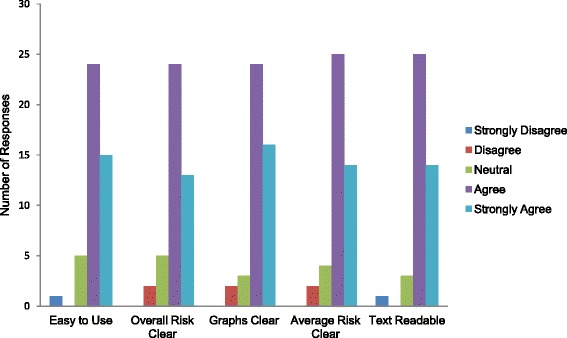
Usability and Clarity of OA Risk Calculator – This figure shows participant responses to six questions about the usability and clarity of OA Risk C. Each of these questions offered responses on a 5 point Likert scale with the following agreement options: “Strongly Disagree,” “Disagree,” “Neutral,” “Agree,” and “Strongly Agree.” The first statement that users rated (“Easy to Use”) was: “The risk calculator was easy to use.” The second statement (“Overall Risk Clear”) was: “My risk of developing knee osteoarthritis was clear and easy to understand.” The third statement (“Graphs Clear”) was: “The graphs showing my risk were clear and easy to understand.” The fourth statement (“Average Risk Clear” was: “My risk of developing knee osteoarthritis compared to the average American was clear and easy to understand.” The fifth statement (“Text Readable”) was: “The text in the risk calculator was easy to read.”

Overall, participants responded favorably to questions about the usability and clarity of the risk calculator. Eighty-seven percent of participants reported agreement with the statement, “The risk calculator was easy to use.” Eighty-four percent agreed that their risk of developing knee osteoarthritis was clear and easy to understand, and 89 % stated that the graphical representation of OA risk was clear and easy to understand (Fig. 7).

Rated ease of use was high across all age, sex, and race/ethnicity groups. Self-reported understanding of OA risk was generally high, ranging from 100 % in Whites to 87 % in Hispanics and 75 % in Blacks. Men reported more difficulty understanding the graphical representation of their OA risk – 17 % of men compared to 3 % of women did not agree that the graphical representation of their risk of developing knee OA was clear and easy to understand.

## Discussion

We have developed an Osteoarthritis Risk Calculator and assessed its ease of use and feasibility in a primary care setting. Overall**,** subjects found that the calculator was clear and that the results were comprehensible. These findings suggest that OA Risk C may be utilized successfully by a wider audience.

Risk calculators have been developed for several conditions, including heart disease, cancer, diabetes, stroke, emphysema, and osteoporosis [18–22]. Many of these risk calculators have been integrated into clinical practice. Recommendations for statin utilization were updated in 2013 to account for calculated risk [34, 35], and the American Heart Association and American College of Cardiology have encouraged public use of their risk calculator. The Carotid Risk Assessment Tool, which allows physicians to view estimated 2-year mortality for patients with carotid artery stenosis, was incorporated into an electronic medical record. Overall, physicians felt that the tool was useful when weighing the risks and benefits of revascularization surgery for patients with carotid artery stenosis and that the tool aided patients in their decision-making [36].

While most risk calculators have been well received by patients, reactions to the results of risk calculators vary. One study showed that diabetic patients using a cardiovascular risk calculator had a diverse range of responses to viewing their risk of experiencing a heart attack and/ or stroke; some reported feeling distressed when their risk was substantially higher than expected [37]. Another study found that in cohort of 50 patients ranging in age from 27 to 84 years old, many had suboptimal understanding of the connection between high cholesterol and cardiovascular disease (CVD) risk. When presented with results from a cardiovascular risk-adjusted age calculator, some study participants felt that their newfound knowledge would motivate positive behavior change [38]. Krones et al. conducted a randomized trial to assess the effect of an intervention including a paper-based CVD risk calculator and information booklet on shared decision-making between patients and physicians in a primary care setting. Compared to the control arm, patients in the intervention group had higher satisfaction and involvement in decision-making [39], indicating that personalized health information may serve to activate patients. Taken together, these previous studies suggest that viewing personalized risk information may lead to a more educated and activated patient population that is motivated for behavior change.

The OA Risk Calculator was well-received overall. Patients reported verbally that learning about their risk of developing OA was helpful and interesting, and the majority of participants believed the calculator was easy to use and that the results were easy to understand. Research suggests that even highly educated individuals are poor at converting between percentages and proportions in the context of risk [40]. Therefore, we used multiple presentations of OA risk, including an icon array form, which shows a pictorial representation of each user’s risk of developing knee OA (Fig. 3).

Primary prevention interventions focused on middle-aged and older populations may have a limited effect due to prolonged exposure to risk factors earlier in life. Educating younger populations about modifiable knee OA risk factors may have an even greater impact by prompting younger persons to minimize their exposure to risk factors. Injury prevention programs have been shown to reduce the number of traumatic knee injuries in sports participants [41–43] and might be more widely used if younger persons were aware that knee injury is a risk factor for OA. Similarly, several weight management interventions developed within the context of OA [13, 44, 45] may be extended to younger persons who are overweight or obese and who wish to lose weight to reduce their risk of developing knee OA.

This study has certain limitations. Because the OAPol model does not contain data for persons younger than 25 or for those identifying as a race/ethnicity other than Black, White, or Hispanic, we were unable to accurately estimate risk for these populations. Only those aged 25 to 45 years were included in this pilot study, and those who identified as a race/ethnicity other than Black, White, or Hispanic were able to participate in the study with the knowledge that the risk information presented would be that of someone with their risk factors who identifies as White. The data used in the OAPol model to generate our risk estimates were derived from multiple data sources. In the analysis we used the best available data. While our current risk ratios are derived from the 2011 report from the Nottingham study, these observations have been confirmed in the most recent meta-analysis published in 2015 [46]. All estimates carry some uncertainty. We minimized uncertainty by conducting a large number of simulations and using data from large cohort studies.

Since the increase in risk due to occupational exposure, history of knee injury, or family history of knee OA was applied to basic incidence rates of non-obese persons, it may lead to some overestimation. The prevalence of these risk factors in the population does not exceed 10 %, so the bias due to overestimation is not substantial. It ranges from 2 % for those with the occupational exposure to 14 % among those with history of knee injury. Additionally, we have assumed that risk factors are independent and multiplicative. If this assumption is not satisfied, the estimated risk among persons with multiple risk factors may be overestimated. To address this limitation, we will include a disclaimer at the footnote of the calculator. Lastly, there is limited information available showing that reducing exposure to risk factors of knee OA will impact risk in a meaningful way. To address this limitation, we carefully described the impact of the risk factor by stating that the comparison is between persons with and without the risk factor. In addition, we will include a disclaimer stating that there is limited data on the reduction of the risk among those who modify a risk factor such as obesity.

Despite these limitations, this study has certain strengths. It is the first to our knowledge that describes the development and acceptability of a risk calculator for osteoarthritis. OA Risk C is unique in its flexibility and adaptability over time. Because it relies on outputs from the OAPol model, it can be updated whenever we update or change the model as new data become available. Moreover, we have illustrated the feasibility and comprehensibility of OA Risk C in a pilot study of 45 subjects in a primary care setting. This suggests that the OA risk calculator may be successfully used by a wider audience. Future research may focus on determining whether or not viewing personalized risk of developing knee OA prompts behavior change to minimize modifiable risk factors.

## Conclusions

We have developed an Osteoarthritis Risk Calculator (OA Risk C) and illustrated its acceptability and feasibility in a pilot study of 45 subjects. This risk assessment tool may be disseminated to a wider audience in order to educate the general population about their personalized risk of developing knee OA and present interventions to minimize exposure to risk factors. Doing so may motivate behavior changes that reduce the incidence and burden of knee OA.

## Abbreviations

OA: Osteoarthritis
OA Risk C: Osteoarthritis risk calculator
OAPol: Osteoarthritis policy
TKR: Total knee replacement
CVD: Cardiovascular disease
